# Haematologic outcomes and associated clinical characteristics among patients receiving Olaparib therapy in the UAE: a retrospective chart review

**DOI:** 10.1080/07853890.2024.2440631

**Published:** 2024-12-13

**Authors:** Lina Wahba, Said Nabil, Saba Kendakji, Mariam Ibrahim, Sham ZainAlAbdin, Salahdein Aburuz, Amal Akour

**Affiliations:** a Department of Clinical Pharmacy, Tawam Hospital, Abu Dhabi Health Services Company, Al Ain, UAE; b Department of Pharmacology and Therapeutics, College of Medicine and Health Sciences, United Arab Emirates University, Al Ain, UAE; c School of Medicine and Health Sciences, George Washington University, Washington, DC, USA; d Department of Biopharmaceutics and Clinical Pharmacy, The School of Pharmacy, The University of Jordan, Amman, Jordan

**Keywords:** Olaparib, anaemia, haematologic indices, transfusion

## Abstract

**Background:**

Poly ADP ribose polymerase (PARP) inhibitors, such as Olaparib (Lynparza^®^), are pivotal in treating certain cancers, particularly those linked to BReast CAncer gene (BRCA) mutations. Despite its established efficacy, Olaparib use is associated with various adverse events (AEs), notably haematologic toxicities, such as anaemia. This retrospective chart review study aimed to examine haematologic outcomes and associated factors in patients treated with Olaparib at a tertiary hospital in the UAE.

**Methods:**

We reviewed the medical charts of patients prescribed Olaparib and focused on haematologic indices at a baseline of 1-month, 3-month and 6-month follow-up periods. Data were analysed to determine the AEs frequency, transfusions need and potential associated patients’ clinical characteristics.

**Results:**

This study included all patients who received Olaparib (*n* = 66). Most patients were females (*n* = 61; 92.4%) and the vast majority were non-smokers (97%) and free of hepatic disease. Themean age of the patients was 57.03-year-old (SD) = 12.06 years), and body mass index (BMI) was 28.16 (SD = 6.40) kg/m^2^. A high rate of anaemia (70.8%) was detected among the patients during their Olaparib therapy. Approximately, one-third of the patients developed neutropenia and thrombocytopenia. Transfusion was needed in almost half of the patients. Glomerular filtration rate (GFR) and neutropenia were significantly correlated with moderate-severe anaemia (OR = 0.097, 95% CI: 0.011–0.88, *p* value = .038) and (OR = 9.04, 95% CI: 1.024–79.78, *p* value = .048), respectively.

**Conclusions:**

Our findings highlight the side effects of Olaparib therapy in terms of haematology which could be avoided. Further studies are needed to better understand the therapeutic management of Olaparib and the mitigation of haematologic complications.

## Introduction

Poly ADP ribose polymerase (PARP) inhibitors, used to treat cancers, have numerous established modes of action, notably base excision repair suppression and PARP entrapment [1]. These mechanisms cause double-stranded breaks to form once the DNA replication forks stall and collapse. Tumours with an evident deficiency in homologous DNA repair (and consequently, in double-stranded break repair) appear to be amenable to PARP inhibitor treatment [2,3]. These include cancers caused by germline or somatic mutations in BReast CAncer gene (BRCA)-1 and BRCA2 [4]. The two primary pathways for mending double-strand DNA breaks are homologous recombination (HR) and nonhomologous end joining (NHEJ). HR is a high-fidelity procedure that takes place mostly during the S and G2 stages of the cell cycle, when a sister chromatid is available. It entails re-sectioning the DSB ends to produce single-stranded DNA, which is subsequently covered with RAD51 to aid strand invasion into the homologous sequence. DNA synthesis occurs with the sister chromatid as a template, and the D-loop is resolved to precisely restore the DNA sequence. In contrast, NHEJ is an error-prone mechanism that occurs throughout the cell cycle and includes direct ligation of DNA ends in the absence of a homologous template. The Ku70/80 complex binds to the DSB, which is then processed by nucleases such as Artemis before being ligated by DNA ligase IV, XRCC4 and XLF. While HR protects genomic integrity by ensuring precise repair, NHEJ is a faster repair pathway that may introduce mutations [5]. Leveraging deficits in repair mechanisms, such as utilizing PARP inhibitors for BRCA-mutated malignancies, is a therapeutic approach.

Olaparib is a bioavailable oral medication that inhibits PARP1 and PARP2 catalytic activities [6]. It is approved for treating advanced ovarian and breast cancers caused by germline BRCA1/2 mutations [4]. It has also been indicated as a maintenance therapy for ovarian cancer after platinum-based chemotherapy, demonstrating that PARP inhibition has benefits beyond tumours with BRCA1/2 mutations [7,8]. Adverse event (AE) tolerance, in addition to maintaining quality of life, is a significant concern when beginning Olaparib treatment in all prospective patients who have previously undergone chemotherapy. Nausea, vomiting, lethargy, anaemia and dysgeusia are common AEs associated with Olaparib that can affect patients’ daily life activities [9–11]. With specific interest, haematologic toxicities, including anaemia, are frequently linked to PARP inhibitors, including Olaparib, according to a meta-analysis of 12 randomized controlled trials (RCTs) [12]. In a meta-analysis of nine controlled trials (*n* = 2074 patients) with advanced ovarian, gastric, prostate, lung or breast cancer, the relative risk of all-grade and high-grade anaemia for the Olaparib-treated group (including combination treatments) versus placebo/control was 2.10 (95% confidence interval (CI): 1.48–2.98) and 3.15 (95% CI: 1.73–5.71), respectively [13]. Different methods have been adopted to mitigate Olaparib toxicities [14], including haematological toxicity. Dose reductions and/or suspensions were mostly utilized where treatment would be suspended for any grade ≥3 toxicity, for instance, and resumed if the toxicity subsided to a grade of ≤1(8). In contrast, blood transfusion should be performed together with dosage reduction if the haemoglobin (Hgb) level drops to less than 7 g/dL [15]. Although randomized clinical trials are likely to report outcomes similar to real-world occurrences, there is a chance that side effects are underreported in these trials because stringent eligibility requirements frequently result in trial participants who are younger and fitter than those in community practice [16].

Therefore, this study aimed to assess haematologic associated outcomes and factors among patients receiving Olaparib therapy in a tertiary hospital in the UAE.

## Methods

### Data settings and subjects

This was a retrospective chart review of all adult male and female patients (≥18-year-old), attending the oncology ward in Tawam hospital, a large tertiary hospital in the UAE between January 2020 and January 2023 who were prescribed Olaparib monotherapy for any indication during the study period for a minimum of one month (*n* = 66). The patients were treated with the currently approved dose of 300 mg (as two 150 mg tablets) BID [17], resulting in a total daily dose of 600 mg. In 2020, the capsule form was administered 400 mg BID, with a total daily dose of 800 mg [18]. Patients who were on erythropoietin-stimulating agents or had missing or incomplete data were excluded. Follow-up data and haematologic indices were compared at baseline, 1-month, 3-months and 6-months. Interventions to correct anaemia were observed at the 3-month follow-up. Data were collected from the patients’ medical charts and included demographic variables such as age, sex, body mass index (BMI) and clinical data such as renal function and comorbidities.

### Ethical considerations

This study adhered to the ethical guidelines outlined in the Declaration of Helsinki and was reviewed and approved by the Tawam Hospital Human Research Ethics Committee (T-HREC), (approval number: MF2058-2022-831). The requirement for informed consent was waived because of the retrospective nature of the study by the same committee. All personal identifiers were removed from the study records and each participant was assigned a unique code. Data were stored electronically on a secure server with access limited to the research team.

### Outcomes

The frequency of AEs and the need for transfusion were reported, as well as haematologic indices, including Hgb, mean corpuscular volume (MCV), mean corpuscular haemoglobin (MCH) and mean corpuscular haemoglobin concentration (MCHC). Anaemia was classified according to the National Cancer Institute (NCI) [19] as follows:

1. Mild: Hgb 10.0 g/dL to lower limit of normal;
2. Moderate: Hgb 8.0–10.0 g/dL;
3. Severe: Hgb 6.5–7.9 g/dL;
4. Life-threatening: Hgb less than 6.5 g/dL.

### Data management and statistical analysis

Since we included all patients taking Olaparib, the sample size calculation was not applicable. Descriptive baseline characteristics and outcomes were reported as numbers and frequencies if they were categorical variables, but continuous variables were described as mean (standard deviation (SD)) or median (interquartile range (IQR)), when appropriate. Repeated measures one-way analysis of variance (ANOVA) was performed to assess any changes in haematologic indices from baseline and then at the 1-, 3- and 6-month follow-up, and the McNemar test was used to test differences in the proportion of categorical variables at the same time points. Spearman’s nonparametric correlation was performed to evaluate the association between continuous variables and Hgb levels at 3-months, in addition to anaemia severity. Logistic regression was performed, and variables with a *p* value <.25 were entered as independent variables in the model. Statistical Package for Social Sciences (SPSS^®^) software (IBM, version 26, Armonk, NY) was used for data entry and analysis. Statistical significance was considered at a threshold of less than .05.

## Results

This study included all patients who received Olaparib (*n* = 66). The participants characteristics and variables are available in Supplementary file 1 (Table 1). Most patients were females (*n* = 61; 92.4%) and the vast majority were non-smokers (97%) and free of hepatic disease. The mean age of the patients was 57.03-year-old (SD) = 12.06 years), and BMI was 28.16 (SD = 6.40) kg/m^2^. Mean Hgb values and haematocrit were 110.94 (g/dL) and 31.75% (SD = 14.71 g/L and 7.07%), respectively. The most common indication for Olaparib was ovarian cancer (*n* = 53, 80.3%), with a median dose of 600 mg (IQR: 600–800) mg for a median of 12 months (IQR: 5–20). The patients self-reported complete adherence. Less than half of the patients required transfusion at some point during their therapy (*n* = 28, 42.4%). The most common intervention to manage AEs was dose reduction (*n* = 24, 36.8%) followed by discontinuation (*n* = 12, 18.2%) [20].

**Table 1.** Post-therapeutic variables at 6-month follow-up^a^.

Variable	Mean	Median	Std. deviation	Percentiles	
				25	75
Hgb (g/L)	111.00	110.00	13.95	102.00	120.25
HCT (%)	32.62	32.50	3.85	30.00	36.00
MCV (fL)	92.78	95.50	16.02	89.35	100.93
MCH (pg)	38.09	32.80	42.63	29.25	35.15
MCHC (g/L)	341.42	340.50	13.48	330.75	352.00
RDW (%)	16.33	15.85	2.74	14.60	17.48
Platelet count (×10^9^)	231.72	228.00	78.57	181.50	280.0
	Frequency	Percentage^b^			
Anaemia					
No	19	29.2			
Yes	46	70.8			
Need for transfusion					
No	38	57.6			
Yes	28	42.4			
Neutropenia					
No	45	69.2			
Yes	20	30.8			
Thrombocytopenia					
No	47	72.3			
Yes	18	27.7			
Secondary acute myeloid leukaemia					
No	63	96.9			
Yes	2	3.1			
GI toxicity					
No	56	86.2			
Yes	9	13.8			
Pneumonitis					
No	64	98.5			
Yes	1	1.5			

Hb: haemoglobin; HCT: haematocrit; MCV: mean corpuscular volume; MCH: mean corpuscular haemoglobin; MCHC: mean corpuscular haemoglobin; RDW: red blood cell distribution width.

^a^ Haematologic indices were available for 50 patients at follow-up.

^b^ Valid percent.

Data on haematological indices were also retrospectively collected at 1-, 3- and 6-month post-therapy. Table 1 summarizes the data at the 6-month time point. Anaemia occurrence at any time during therapy was also recorded. Approximately, 70% of patients develop anaemia at some time during therapy. Approximately, one-third of patients developed neutropenia and thrombocytopenia. One patient (1.6%) developed secondary acute myeloid leukaemia (AML) at follow-up.

Repeated measures ANOVA was performed to assess any changes in haematologic indices from baseline and then at the 1-, 3- and 6-month follow-up time intervals. MCV was significantly increased at 3-month post follow-up baseline (mean difference = 3.10, *p* = .004). MCH also increased significantly at all follow-up times when compared to baseline (mean differences = 0.63, 1.35 and 1.85; *p* values of .047, .005 and .002, respectively). However, there were no significant changes in the other parameters, including Hgb, HCT and MCHC. Anaemia status was further stratified into mild and moderate-severe anaemia based on the NCI definition (see section ‘Methods’). The McNemar test for paired proportions showed no statistically significant differences between proportions at all follow-up points, but there was a trend of increase in the proportion of patients with moderate-severe anaemia with time (pre-intervention) as follows: 27%, 32.8% and 37.7% at baseline, and at the 1-month and 3-month follow-up points, respectively. To elucidate the patients’ clinical characteristics that might affect the likelihood of more severe anaemia, a Chi-square test was first performed to assess the bivariate associations between anaemia severity at the 3-month follow-up and patients’ categorical characteristics (Supplementary file 2, Table 2). It was found out that glomerular filtration rate (GFR) and neutropenia were significantly associated with anaemia severity. Hence, patients who developed neutropenia and had a lower GFR (<60 mL/min) were more likely to have moderate-to-severe anaemia. Spearman’s nonparametric correlation was performed to evaluate the association between continuous variables and Hgb levels at 3-months, in addition to anaemia severity (Table 2). Age, weight and BMI were significantly associated with Hg levels at the 3-month follow-up, as follows: *r* = −0.28, *p* = .04; *r* = 0.38, *p* < .001; and *r* = 0.37, *p* = .01, respectively, but age was not related to anaemia severity. Logistic regression was performed, and variables with a *p* value <.25 were entered as independent variables in the model. The model fit results showed a −2-log likelihood of 38.27, and 55.7% of variability in anaemia severity was accounted for in the model. However, only neutropenia and GFR were correlated with anaemia severity after accounting for all potentially significant covariates, that is, developing neutropenia, and lower GFR was associated with a higher likelihood of developing severe anaemia (Table 3).

**Table 2.** Correlation of continuous variables with haemoglobin and anaemia severity at 3-month follow-up.

Variable	Hgb levels (g/L)		Anaemia severity	
	*r*	*p* Value	*r*	*p* Value
Age (years)	−0.28*	.04	0.26	.06
Weight (kg)	0.38**	<.001	−0.37**	.01
BMI (kg/m^2^)	0.37**	.01	−0.35*	.01
Performance status grade	−0.09	.55	0.02	.90
ALT (U/L)	0.24	.09	−0.23	.11
AST (U/L)	0.09	.54	−0.01	.97
Bilirubin direct (µmol/L)	−0.26	.12	0.04	.83
WBC (×10^9^)	0.13	.37	−0.11	.45
RBC (×10^9^)	0.30*	.03	−0.16	.26
Hgb (g/L) at baseline	0.50**	<.001	−0.44**	<.001
HCT (%) at baseline	0.47**	<.001	−0.39**	<.001
MCV (fL) at baseline	0.07	.64	−0.24	.09
MCH (pg) at baseline	0.10	.48	−0.29*	.04
MCHC (g/L) at baseline	0.22	.12	−0.25	.07
RDW (%) at baseline	−0.16	.28	0.15	.31
Platelet (×10^9^) count at baseline	0.07	.65	0.05	.73
Dose (mg)	0.08	.56	−0.004	.76
Duration (months)	0.02	.90	−0.008	.60

BMI: body mass index; ALT: alanine aminotransferase; AST: aspartate aminotransferase; WBC: white blood cells; RBC: red blood cells; Hgb: haemoglobin; HCT: haematocrit; MCV: mean corpuscular volume; MCH: mean corpuscular haemoglobin; MCHC: mean corpuscular haemoglobin; RDW: red blood cell distribution width. *p<0.05; **p<0.001.

**Table 3.** Logistic regression between patient characteristics and likelihood of severe anaemia.

Variable	*p* Value	OR	95% CI	
			Lower	Upper
Age	.777	1.012	0.930	1.103
Gender, male = 0, female = 1 (1)	.541	0.217	0.002	29.05
Neutropenia on follow up No = 0, yes = 1 (1)	.038	0.097	0.011	0.879
GFR_Category (1)	.048	9.038	1.024	79.776
BMI	.785	0.982	0.859	1.121
ALT (U/L)	.123	0.907	0.801	1.027
Transfusion (1)	.450	1.888	0.363	9.813
Indication (1)	.952	0.906	0.036	23.088

Patients’ clinical characteristics that are related to blood transfusion were also evaluated. As shown in Table 4, continuous variables were compared using independent *t*-test or nonparametric equivalent Mann–Whitney’s test, as indicated. Compared to the patients who did not require transfusion, the mean age was significantly higher in those who required transfusion (60.46 (12.76) vs. 54.50 (11.02) year-old; *p* = .046), but BMI was significantly lower (26.24 (6.06) vs. 29.64 (6.34) kg/m^2^; *p* = .037). The mean MCV and MCH values were significantly lower in those in patients who underwent transfusion at the 3-month follow-up (87.11(15.64) vs. (95.96 (8.19) %; *p* = .012) and (29.94(3.62) vs. (33.24 (3.09) pg; *p* < .001); respectively. Similarly, MCHC was significantly lower in those with transfusion requirements both at baseline (332.22 (12.92) vs. 341.67 (10.10) g/L; *p* = .002) and 6-month follow-up (336.80 (13.81) vs. 344.50 (12.56); *p* = .047). Regarding categorical variables, however, the Chi-square test showed no significant difference between the proportions of patients according to transfusion requirement (data not shown).

**Table 4.** Comparing demographic and clinical characteristics by transfusion need.

Variable	Need for transfusion						
	Yes			No			
	*N*	Mean	SD	*N*	Mean	SD	*p* Value
Age (years)	28	60.46	12.76	38	54.50	11.02	.046*
BMI (kg/m^2^)	26	26.24	6.06	37	29.64	6.34	.037*
ALT (U/L)	24	17.71	11.20	37	24.32	23.72	.207
AST (U/L)	24	27.46	20.63	37	27.32	19.64	.980
Bilirubin direct (µmol/L)	16	3.50	1.21	28	3.26	1.84	.645
WBC (×10^9^)	25	6.84	4.19	36	6.29	1.85	.490
RBC (×10^9^)	25	4.61	6.14	36	3.62	.72	.545
Hgb (g/L) at baseline	27	109.81	14.75	36	111.78	14.84	.604
Hgb (g/L) at 1 month	25	104.36	17.64	33	109.64	14.17	.212
Hgb (g/L) at 3 months	26	101.77	20.47	27	111.41	18.05	.075
Hgb (g/L) at 6 months	20	106.40	16.04	30	114.07	11.67	.056
HCT (%) at baseline	28	31.75	7.23	37	31.76	7.05	.997
HCT (%) at 1 month	26	28.66	9.85	35	30.09	8.63	.551
HCT (%) at 3 months	27	29.21	8.21	28	31.00	8.05	.417
HCT (%) at 6 months	21	30.29	8.01	30	33.17	3.64	.089
MCV (fL) at baseline	27	89.02	6.40	36	89.78	8.01	.688
MCV (fL) at 1 month	25	87.96	6.33	33	90.92	8.72	.157
MCV (fL) at 3 months	26	87.11	15.64	27	95.96	8.19	.012*
MCV (fL) at 6 months	20	93.16	7.29	30	92.52	19.96	.891
MCH (pg) at baseline	27	29.55	2.74	36	30.69	3.14	.137
MCH (pg) at 1 month	25	29.42	2.76	33	31.15	3.12	.032*
MCH (pg) at 3 months	26	29.94	3.62	27	33.24	3.09	<.001*
MCH (pg) at 6 months	20	45.88	67.47	30	32.90	4.24	.296
MCHC (g/L) at baseline	27	332.22	12.92	36	341.67	10.10	.002*
MCHC (g/L) at 1 month	25	321.42	61.79	33	342.45	10.38	.059
MCHC (g/L) at 3 months	26	322.11	63.53	27	346.63	10.06	.053
MCHC (g/L) at 6 months	20	336.80	13.81	30	344.50	12.56	.047*
RDW (%) at baseline	27	16.00	3.04	36	16.27	2.97	.728
RDW (%) 1 month	25	15.94	2.84	33	16.44	2.64	.499
RDW (%) at 3 months	26	17.57	2.87	27	16.50	2.18	.134
RDW (%) at 6 months	20	16.91	3.59	30	15.94	1.97	.224
Platelet (×10^9^) count at baseline	27	208.94	74.09	36	238.61	86.64	.158
Platelet (×10^9^) count at 1 month	25	190.34	72.90	33	226.21	83.57	.093
Platelet (×10^9^) count at 3 months	26	205.50	91.95	27	210.15	68.40	.835
Platelet (×10^9^) count at 6 months	20	220.14	81.38	30	239.43	77.06	.401
Dose (mg)	28	657.14	150.13	38	668.42	125.43	.741
Duration of therapy (months)	27	13.06	10.86	36	16.08	15.83	.397

BMI: body mass index; ALT: alanine aminotransferase; AST: aspartate aminotransferase; WBC: white blood cells; RBC: red blood cells; Hgb: haemoglobin; HCT: haematocrit; MCV: mean corpuscular volume; MCH: mean corpuscular haemoglobin; MCHC: mean corpuscular haemoglobin; RDW: red blood cell distribution width.

* Statistically significant.

## Discussion

In this study, we investigated the haematologic outcomes and potentially associated patients’ clinical characteristics among patients receiving Olaparib therapy, focusing on their impact on anaemia and other haematological parameters. The results highlight several important observations that have implications for the management of patients undergoing this treatment.

One of the key findings was the high rate of anaemia, with 70.8% of the patients experiencing it at some point during Olaparib therapy, which was higher than that observed in previous studies. A RCT by Mirza et al. [21], which included 138 patients treated with niraparib, showed that anaemia of any grade occurred in 50.1% of patients. Furthermore, our study showed that approximately one-third of the patients developed neutropenia and thrombocytopenia, comparable to the study by Mirza et al. [21]. Notably, one patient in our study developed AML, a rare but serious AE of Olaparib. Recent large pharmacovigilance studies have shown an association between Olaparib and AML, especially with long-term use [22]. Zhao et al. [22], showed that the median time to onset of AML while on PARP inhibitor, including Olaparib was 355 days (∼11.7 months), with Olaparib having the strongest association with AML. Consistent with our data, a study of 32,356 patients treated for any gynaecological cancer with PARP inhibitors showed that the rate of secondary MDS/AML in the total population was almost 1% but was nine times higher in ovarian cancer patients. This rate was higher in patients with thrombocytopenia or co-administration of carboplatin [23]. These findings highlight the need for careful monitoring of patients on long-term Olaparib therapy for the development of fatal AEs.

The need for transfusions in almost half of the patients underscores the clinical significance of these haematological changes. The follow-up duration in our study was 6 months, shorter than that RCT by Pujade-Lauraine et al. [7]undefined; however, they reported fewer anaemia events than ours (19% of 195 patients), similar to the trial by Colombo et al. [24]. Differences in percentages may reflect variations in the study design, monitoring frequency and patient characteristics at baseline, such as pre-existing comorbidities or variations in Olaparib dosing regimens. The follow-up data at 6 months post-therapy showed that certain haematologic indices, such as MCV and MCH, had increased, suggesting alterations in red blood cell morphology over time. Although Hgb, HCT and MCHC did not change significantly, the trends in MCV and MCH may indicate macrocytic anaemia due to folic acid deficiency [25]. This type of anaemia is characterized by larger-than-normal red blood cells and could explain the high rates of anaemia observed. Nevertheless, a more recent retrospective study reported macrocytic anaemia in 50% of 18 patients with anaemia on Olaparib, but this was not correlated with vitamin B12 or folic acid deficiency [26]. It is essential to differentiate whether these changes resulted from macrocytic anaemia or were an effect of the intervention observed at the 3-month follow-up, as it is difficult to delineate both interpretations, especially that MCV, MCH and MCHC remained within normal limits.

The haematologic indices in patients who received the intervention at 3-month showed a significant increase in MCV and MCH compared to the control group, suggesting a potential effect of the intervention on red blood cell morphology. It is noteworthy that other factors should be considered, such as patient characteristics, concurrent medications and underlying medical conditions. Considering these variables is important to elucidate whether the observed changes were due to the intervention or to other factors.

Therefore, to better understand the factors contributing to anaemia severity, we conducted stratification and logistic regression analysis. The results indicated that neutropenia and lower GFR were significantly associated with moderate-to-severe anaemia. This is an important finding as it suggests that patients with these risk factors may require more careful monitoring and potentially earlier intervention to prevent severe complications. A recent small retrospective study also reported that baseline neutropenia was a predictor of Olaparib discontinuation due to haematologic side effects following 3 months of therapy [27]. Indeed, the levels of Olaparib are significantly increased in patients with renal failure, which increases toxicities including anaemia [28]. Interestingly, a recent pharmacokinetic/pharmacodynamic model from real-word data showed that there is a target Olaparib concentration (3500–4000 ng/mL) to prevent the risk of anaemia [29], which is also a function of creatinaemia. Thus, linking the levels of drug with the onset of kidney failure would help tailor individualized drug dose adjustment [29]. In contrast, Tashiro et al. [30] showed that a low baseline RBC count, HCT and Hgb levels, in addition to BRCA1/2 mutation, were significantly associated with the onset of grade ≥3 anaemia. Our study almost conformed to these findings in the bivariate analysis, as baseline Hgb, HCT, MCV, MCH and MCHC were correlated with anaemia severity; however, this significance disappeared in the multivariate logistic regression model. In addition, the baseline MCHC levels were lower in patients who underwent transfusion at the 3-month follow-up. These differences might be attributed to the multicentre nature of Tashiro et al. study, larger sample size, and inclusion of patients who only took Olaparib for 90 days. Moreover, unlike our study, data on renal function, liver function or other AEs were not collected; therefore, the effect of these covariates could not be evaluated in the latter study. Correlation analysis revealed that certain variables, including age, weight and BMI, were significantly correlated with Hgb levels at the 3-month follow-up. However, age was not related to anaemia severity, so while age may influence Hgb levels, it may not be a strong predictor of anaemia severity. A Japanese study, by Shiraishi et al. [31] showed that anaemia was significantly correlated with lower body weight. However, Stanisławiak-Rudowicz et al. [32] showed a more severe haematologic side effects after one month of Olaparib therapy in overweight and obese patients indicated by lower Hgb and RBC values.

Lower weight and BMI were found to be correlated with anaemia severity and transfusion requirement in our study; however, as previously mentioned, the effect was not retained in further analysis, as the logistic regression analysis demonstrated that only neutropenia and GFR were significant predictors of anaemia severity after accounting for other potentially significant covariates. These results highlight the importance of assessing renal function and monitoring WBC counts at baseline and during Olaparib therapy, as these factors may play a crucial role in predicting severe anaemia.

## Study limitations and future directions

Despite the valuable insights gained from this study, it has several limitations. The relatively small sample size and retrospective design could have introduced bias or confounding factors that were not fully accounted for. Most of our samples were females, which can introduce selection bias, as they are more prone to anaemia than males. However, this is inevitable as most of our study samples comprised of ovarian and breast cancer patients [33]. Also, the follow-up time was limited to 6 months, which can be a potential limitation of the study because some side effects can start to show after this period of time. Additionally, the study did not consider other variables such as patient comorbidities, concurrent medications or lifestyle factors such as nutrition that might affect haematologic outcomes.

## Conclusions

In conclusion, this study sheds light on the haematologic outcomes associated with Olaparib therapy and identifies the key risk factors for severe anaemia. These findings suggest that clinicians should monitor patients closely for signs of neutropenia and reduced GFR, as these factors are associated with a higher likelihood of developing moderate-to-severe anaemia. This knowledge can inform more personalized treatment approaches and improve outcomes in patients undergoing Olaparib therapy. Future research should address these limitations by conducting prospective studies with larger sample sizes, including more males. More comprehensive data collection is warranted to assess the effect of other confounders on anaemia and its severity, namely nutritional status. Baseline iron, vitamin B12 and folic acid should be evaluated at baseline and at follow-up. Furthermore, longitudinal studies that track patients over long-term follow-up could provide deeper insights into the progression of haematological changes during Olaparib therapy.

## Supplementary Material

Supplemental Material

## Data Availability

Data are available upon request from the principal investigator.
