# Supplementary material for: Haematologic outcomes and associated clinical characteristics among patients receiving Olaparib therapy in the UAE: a retrospective chart review
Source: Ann Med. 2024 Dec 13;57(1):2440631. doi: 10.1080/07853890.2024.2440631 (PMC11648135; doi:10.1080/07853890.2024.2440631)
Supplement: Supplemental Material [file IANN_A_2440631_SM9638.zip › Supplementary_file/Supplementary_file_1.docx]

**Supplementary file 1**

**Table 1**

***Table 1: Participants characteristics and variables (n=66)***

| Variable | **Mean** | **Median** | **SD** | **Percentiles** | |
| --- | --- | --- | --- | --- | --- |
|  |  |  |  | **25** | **75** |
| Age (years) | 57.03 | 58.50 | 12.06 | 47.00 | 65.00 |
| BMI (Kg/m^2^) | 28.24 | 28.16 | 6.40 | 23.26 | 31.72 |
| Performance status grade | 0.47 | 0.00 | 0.90 | 0.00 | 1.00 |
| ALT (U/L) | 21.72 | 17.00 | 19.91 | 12.00 | 22.50 |
| AST (U/L) | 27.38 | 21.00 | 19.86 | 17.00 | 30.50 |
| Bilirubin Direct (µmol/L) | 3.35 | 2.95 | 1.63 | 2.20 | 4.18 |
| WBC (x10^9^) | 6.52 | 5.80 | 3.02 | 4.60 | 7.65 |
| RBC (x10^9^) | 4.02 | 3.51 | 3.95 | 3.06 | 4.07 |
| Hgb (g/L) | 110.94 | 110.00 | 14.71 | 99.00 | 124.00 |
| HCT (%) | 31.75 | 33.00 | 7.07 | 29.00 | 36.00 |
| MCV (fL) | 89.45 | 89.50 | 7.31 | 84.20 | 93.90 |
| MCH (pg) | 30.20 | 30.60 | 3.01 | 28.00 | 31.80 |
| MCHC (g/L) | 337.62 | 339.00 | 12.24 | 332.00 | 345.00 |
| RDW (%) | 16.15 | 15.50 | 2.978 | 13.70 | 17.90 |
| Platelet Count (x10^9^) | 225.89 | 213.00 | 82.21 | 173.00 | 261.00 |
| Total daily dose of Olaparib (mg) | 663.64 | 600.00 | 135.46 | 600.00 | 800.00 |
| Duration of Olaparib therapy (months) | 14.62 | 12.00 | 13.85 | 5.00 | 20.00 |
|  | **Frequency** | **Percentage** | | | |
| Gender  Male  Female | 5  61 | 7.6  92.4 | | | |
| Smoking status  No  Former  Yes | 64  1  1 | 97.0  1.5  1.5 | | | |
| Renal function  Stage 1  Stage 2  Stage 3 and more | 37  14  10 | 60.7*  23.0  16.4 | | | |
| Hepatic disease (% No) | 66 | 100 | | | |
| BRCA mutation  Negative  Positive  N/A | 13  47  4 | 20.3*  73.4  6.3 | | | |
| Olaparib indication  Ovarian cancer  Prostate cancer  Breast Cancer  Others | 53  7  5  1 | 80.3  10.6  7.6  1.5 | | | |
| Type for therapy adjustment  No adjustment  Dose reduction  Withholding  Discontinuation  Dose reduction then withholding  Dose reduction then discontinuation | 25  24  2  12  2  2 | 37.9  36.4  3.0  18.2  3.0  3.0 | | | |

BMI, body mass index; ALT, alanine aminotransferase; AST, aspartate aminotransferase; WBC, white blood cells; RBC, red blood cells; Hgb, hemoglobin; HCT, hematocrit; MCV, mean corpuscular volume; MCH, mean corpuscular hemoglobin; MCHC, mean corpuscular hemoglobin; RDW, red blood cell distribution width. *Valid percent.
