# Supplementary material for: Haematologic outcomes and associated clinical characteristics among patients receiving Olaparib therapy in the UAE: a retrospective chart review
Source: Ann Med. 2024 Dec 13;57(1):2440631. doi: 10.1080/07853890.2024.2440631 (PMC11648135; doi:10.1080/07853890.2024.2440631)
Supplement: Supplemental Material [file IANN_A_2440631_SM9638.zip › Supplementary_file/Supplementary_file_2.docx]

Supplementary file 2

Table 2

**Table 2: Patients characteristics in relation with anemia severity at 3-month follow-up treatment (n=63).**

| **Variable** | **N (%)** | | **P-value*** |
| --- | --- | --- | --- |
|  | **Mild**  **(n=33)** | **Moderate to severe**  **(n=20)** |  |
| **Gender**  **Male**  **Female** | 1 (3)  32 (97) | 3 (15)  17 (85) | 0.11 |
| **Smoking**  **No**  **Yes**  **Former** | 33 (100)  0 (0)  0 (0) | 18 (90)  1 (5)  1 (5) | 0.18 |
| **Hepatic disease**  **No** | 33 (100) | 20 (100) | -- |
| **BRCA mutation**  **Negative**  **Positive**  **Not available** | 5 (15.6)  24 (75)  3 (9.4) | 5 (26.3)  14 (73.7)  0 (0.0) | 0.290 |
| **Need for transfusion**  **No**  **Yes** | 20 (60.6)  13 (39.4) | 7 (35.0)  13 (65.0) | 0.071 |
| **Olaparib indication**  **Ovarian cancer**  **Others** | 28 (84.8)  5 (15.2) | 16 (80.0)  4 (20.0) | 0.216 |
| **GFR (ml/min)**  **≥ 60**  **<60** | 28 (84.8)  5 (15.2) | 12 (60.0)  8 (40.0) | 0.042 |
| **Need of therapy adjustment**  **No**  **Yes** | 10 (30.3)  23 (69.7) | 8 (40.0)  12 (60.0) | 0.470 |
| **Neutropenia**  **No**  **Yes** | 19 (57.6)  14 (42.4) | 17 (85)  3 (15) | 0.038 |
| **Thrombocytopenia**  **No**  **Yes** | 20 (60.6)  13 (39.4) | 17 (85)  3 (15) | 0.061 |
| **Secondary AML**  **No**  **Yes** | 31 (93.9)  2 (6.1) | 20 (100)  0 (0.0) | 0.262 |
| **GI toxicity**  **No**  **Yes** | 29 (87.9)  4 (12.1) | 28 (90.0)  2 (10) | 0.813 |

AML: Acute myeloid leukemia, GFR: Glomerular filtration rate, GI: gastrointestinal.

*Chi-square
